# Misplaced Right Internal Jugular Catheter Mimicking the Track of the Left Internal Jugular Catheter

**DOI:** 10.7759/cureus.91313

**Published:** 2025-08-30

**Authors:** Mahendra Atlani, Athulya Thekkedathu Harukumar, Sai T Molakapuri, Abhishek Singhai, Mahadev Meena, Aman Kumar, Vaibhav Ingle

**Affiliations:** 1 Nephrology, All India Institute of Medical Sciences Bhopal, Bhopal, IND; 2 Medicine, All India Institute of Medical Sciences Bhopal, Bhopal, IND; 3 General Medicine, All India Institute of Medical Sciences Bhopal, Bhopal, IND; 4 Radiology, All India Institute of Medical Sciences Bhopal, Bhopal, IND

**Keywords:** carotid puncture during ijv cannulation, complications of internal jugular vein cannulation, left ijv cannulation, misplaced internal jugular catheter, persistent left superior vena cava (plsvc), vascular anomalies central venous catheter

## Abstract

A 59-year-old female with end-stage kidney disease was placed on a temporary double-lumen catheter in the right internal jugular vein (IJV) at the community dialysis center. After encountering a blood flow issue during the third hemodialysis (HD), she was referred to a tertiary care hospital for conversion to a tunneled catheter. An X-ray of the chest revealed an atypical position of the catheter. It was crossing the mediastinum to the left at the level of the carina, almost like a mirror image of the usual left IJV catheter track. The possibilities were congenital anomalies of the right IJV, persistent left superior vena cava, or inadvertent placement in any other vessel. The CT angiogram revealed that the catheter was in the arch of the aorta. The catheter was safely removed without any complications (i.e., bleeding, thrombosis, or embolism). This case demonstrates that if, on an X-ray, right IJV vascular access appears to be crossing the mediastinum to the left, resembling a mirror image of left IJV placement, it may be located in the arch of the aorta. It is likely that the placement was not image-guided, and the backflow check was not interpreted.

## Introduction

Vascular access procedures are associated with various risks. These risks depend upon the performer's experience, use of image guidance, and the patient’s vascular anatomy. The tip of the placed catheters should reach the junction of the superior vena cava and the right atrium [1]. Catheters placed in the left-sided internal jugular vein (IJV) should reach the superior vena cava via the left brachiocephalic vein; therefore, the left IJV catheter track is longer and, on X-ray, appears to run from left to right. In contrast, a right IJV catheter has a straighter course as it joins the right subclavian vein and opens into the superior vena cava via the right brachiocephalic vein, so a catheter placed in the right IJV appears only on the right side of the mediastinum on X-ray.

Various complications of IJV cannulation include carotid artery puncture, pseudoaneurysms, arteriovenous fistulas, subclavian (0.1-1%) artery injury leading to hemothorax (1%), and vertebral artery injuries (0.2%) [2]. Perforation of the aorta can also occur, and cardiac tamponade can result if the cannula-site perforation is within the pericardial reflection [3,4].

Here we describe a case where right IJV cannulation resulted in carotid artery puncture and placement of a catheter into the arch of the aorta via the common carotid artery. Our main objective was to present its radiographic appearance, further diagnostic workup, and management.

## Case presentation

A 59-year-old female with end-stage kidney disease (ESKD) was referred for malfunction of a temporary double-lumen hemodialysis catheter that was placed in the right IJV at the community dialysis center by a general physician three days ago. During the third dialysis session at the community dialysis center, a blood flow problem was noted, and the patient was referred for conversion to a tunneled vascular catheter. She was diagnosed with chronic kidney disease due to chronic glomerulonephritis six months ago and was under conservative treatment, but her disease progressed to ESKD, and she was planned to initiate hemodialysis.

There was no history of previous cannulations of the right IJV or use for chemotherapy infusion through the vein. Patient was comfortable and complained about mild pain at the site of catheter insertion only. There was no swelling around the insertion site, and no abnormal dilatation of blood vessels was seen on the left side of the neck. Blood pressure was well within the acceptable range of 140/90 mmHg, SpO_2_ was 99% on room air, and no pallor or pedal edema was noted. Heart sounds were heard normally on the left side of the sternum at the normal position. The rest of the systemic examination was also within normal limits.

Investigations

**Figure 1:**
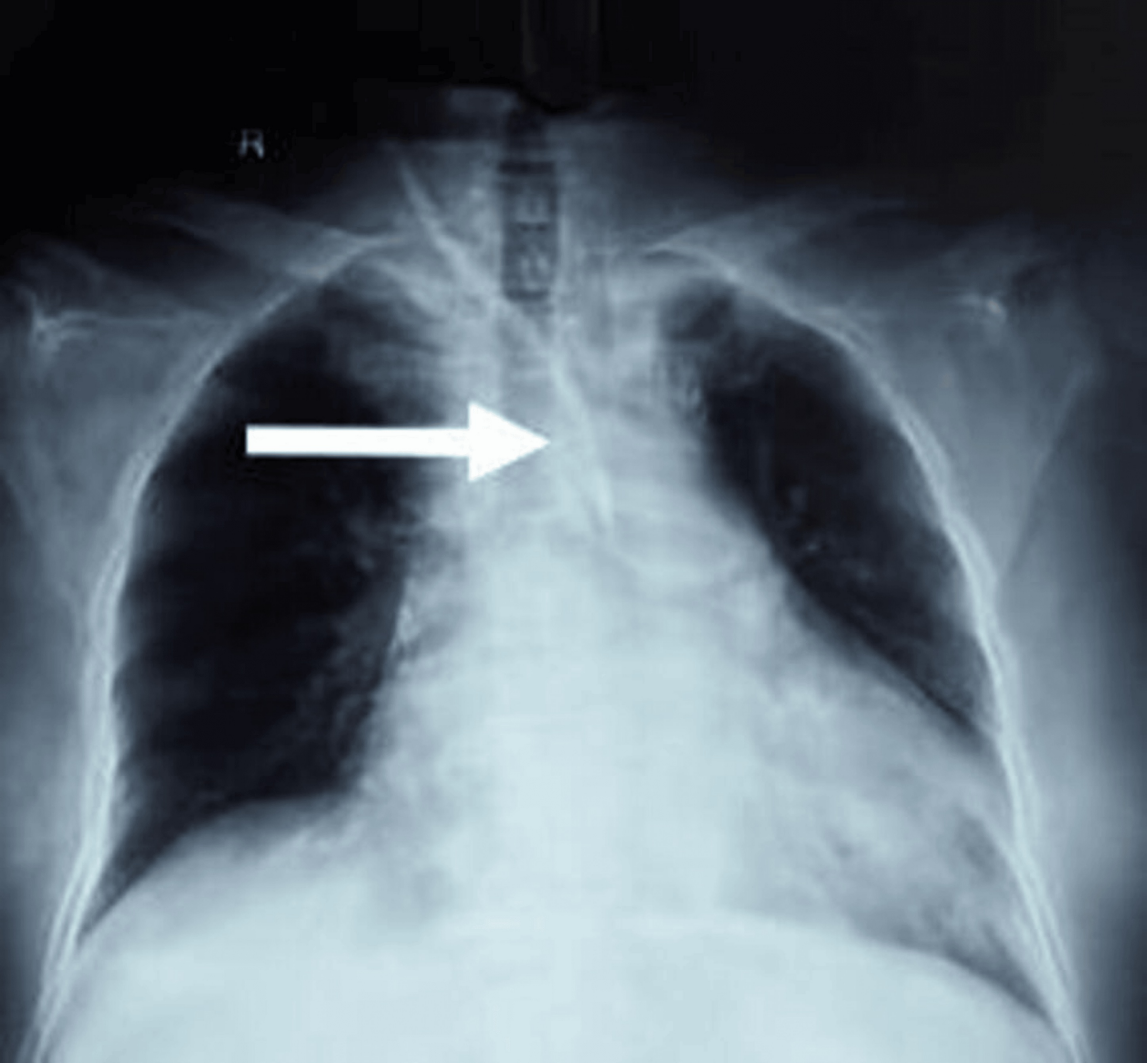
Misplaced right IJV catheter. Arrow displaying atypical position of right IJV catheter - crossing to the left of the mediastinum and mirroring the left IJV catheter track. IJV: internal jugular vein

**Figure 2:**
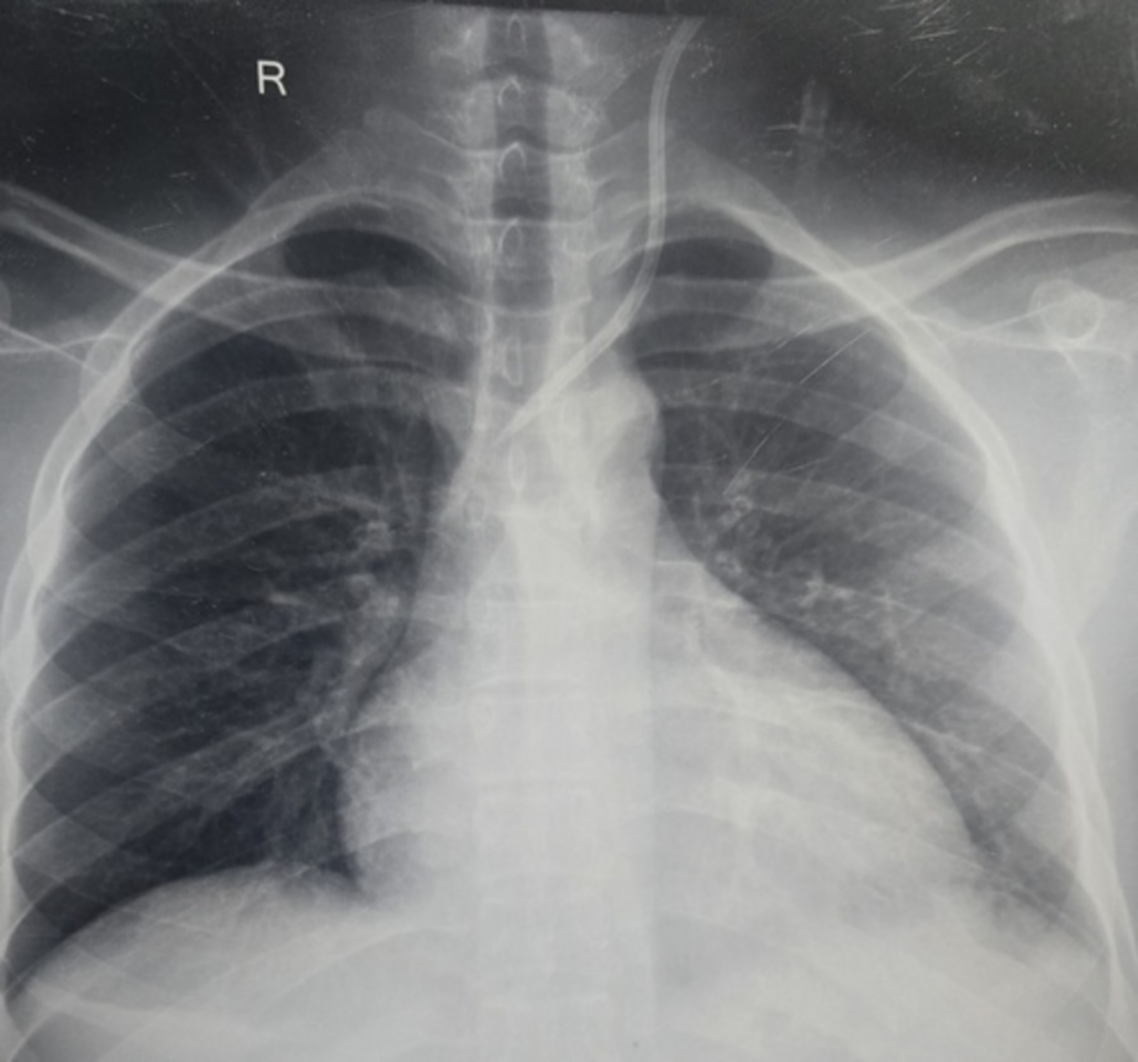
X-ray showing normal course of left IJV catheter. IJV: internal jugular vein

**Figure 3:**
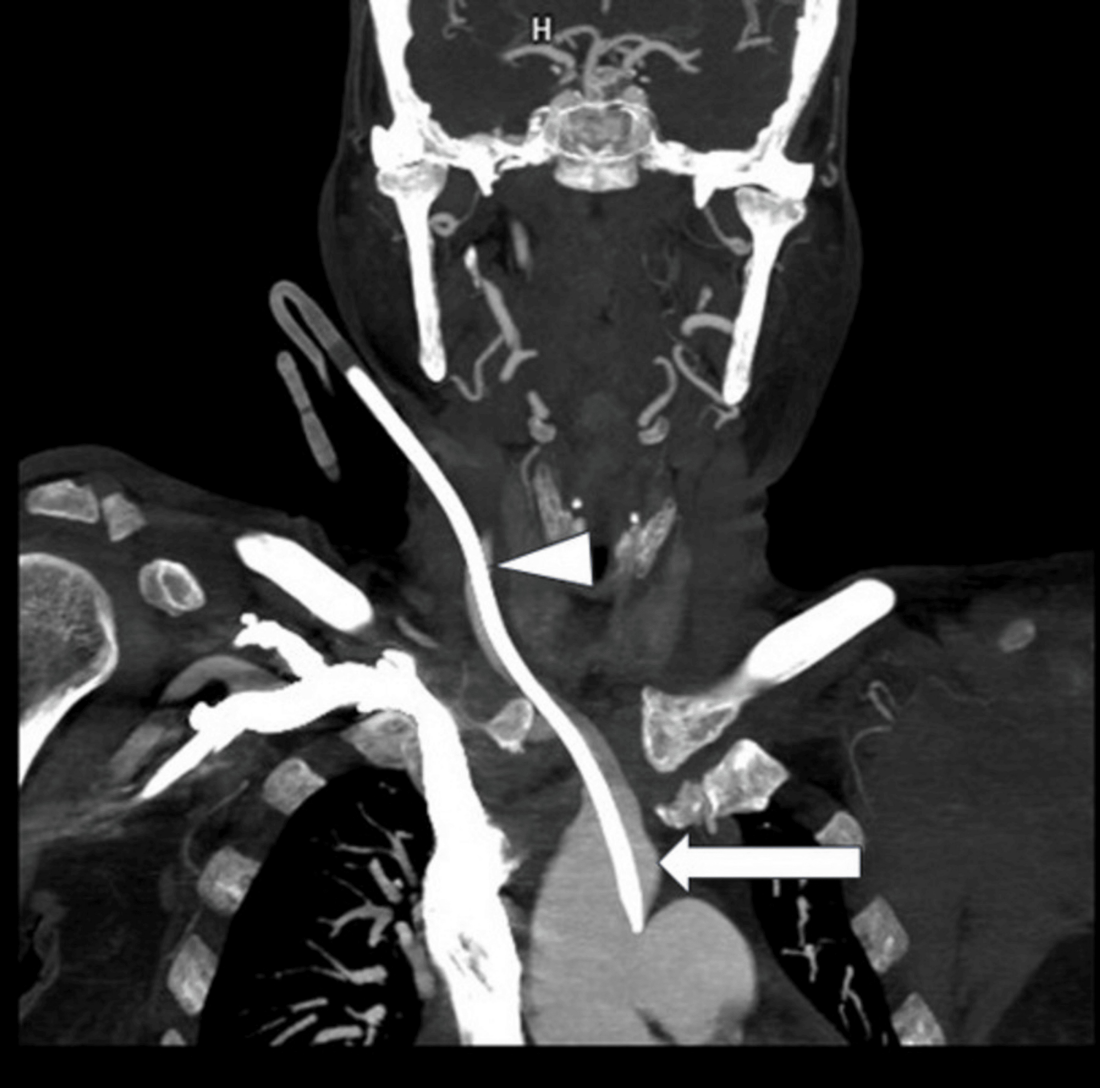
Coronal MIP-reconstructed CT angiography image showing the dialysis catheter in the right common carotid artery (arrowhead), extending up to the aortic arch (arrow). MIP: maximum intensity projection

The patient's hemoglobin (Hb), platelet count, and coagulation parameters were normal; however, renal function was deranged, but not requiring urgent dialysis (Table 1). A routine chest X-ray was obtained to see the catheter position before removal and planned exchange over a guide wire. The X-ray image revealed an atypical position of the catheter, crossing the mediastinum to the left at the level of the carina (Figure 1), almost like a mirror image of the usual left IJV catheter track (Figure 2). A contrast tomographic (CT) angiogram was planned, which revealed the catheter to be in the arch of the aorta, passing through the right common carotid artery (Figure 3). There was no thrombus around the catheter, no collection, hematoma, or any other serious pathology such as pneumothorax.

**Table 1:** Laboratory reports of lab parameters carried out.

Lab parameters	Results	Reference values
Hemoglobin	10 g/dL	12-16 g/dL or 120-160 g/L
Platelets	150,000/µL	150-450x10^9^/L or 150,000-450,000/µL
International normalized ratio (INR)	1.1	0.8-1.1
Blood urea nitrogen (BUN)	62 mg/dL	6-24 mg/dL or 2.1-8.5 mmol/L
Serum creatinine	4 mg/dL	0.59-1.04 mg/dL or 52.2-91.9 µmol/L

Differential diagnosis

The clinical possibilities that were considered were congenital anomalies of the right IJV - absent right IJV after origin with possible drainage to the left IJV via the anterior jugular vein, inadvertent placement in another vessel, persistent left superior vena cava, or even extravascular position of the tip [5]. Contrast tomography angiography showed the catheter to be in the common carotid artery and extending to the aortic arch, thereby ruling out other possibilities.

Treatment

The imperative of removal and possible risk of extravasation were assessed. Endovascular treatment, which involves angiography and stent placement to address arterial damage and repair the vessel, was dropped in favor of open surgical treatment after assessing the time elapsed, the artery involved, and the expertise available. The catheter was pulled out in the operating room, with a ready backup for open surgical intervention and repair if required. However, it was safely removed without any complications (i.e., bleeding, embolism, or vessel thrombosis). A post-removal ultrasound Doppler of the right carotid and common carotid arteries did not reveal pseudoaneurysm, carotid-jugular fistula, thrombosis, or hematoma.

Follow-up

Patient remained stable post removal of catheter; no neurological deficit or shock developed. A left-sided cuffed vascular catheter was placed, and hemodialysis was continued through it. The patient remained under treatment for three weeks; thereafter, she suffered a cardiac arrest at home and expired.

## Discussion

Right or left IJV access placement is associated with various risks. Complications are more with procedures on the left side compared to the right because of the straighter course of the right IJV. However, this report is about the complications of a catheter placed in the right IJV.

Vascular access procedures are associated with various risks. Carotid artery puncture is a common complication, occurring in approximately 6-25% of cases when landmark-guided IJV catheterization is performed [6,7]. This usually occurs in cases where IJV overlaps the CA [6]. Uncontrollable bleeding or hematoma may occur in 40% of the cases [7]. This, in conjunction with manual pressure, can lead to cerebrovascular neurologic deficit (27%) and death (20-40%) [8].

Once a carotid artery is punctured and the performer fails to pay attention to the bright red color of aspirate or spontaneous push back of the plunger, by further maneuver, the catheter can get placed in any of its continuation, i.e., common carotid artery, innominate artery, or the arch of the aorta [9]. Once a catheter is placed, a high SpO_2_ of aspirated blood can also confirm its arterial position. It is interesting to note that, although the incidence of carotid puncture is higher (6-25%), carotid artery cannulation by a large-bore catheter is a rare occurrence, occurring in only 0.1-0.5% of cases [10]. The remaining percentage refers to cases where the arterial puncture was noticed in time, and proper measures were taken, and as such, no accidental carotid cannulation occurred. In the present case, also, probably the carotid puncture went unnoticed, and the artery got cannulated.

Performing IJV cannulation under ultrasound (USG) guidance can help avoid these complications. One study found that USG guidance was useful in avoiding carotid puncture while cannulating the right IJV on the first attempt, especially in pediatric patients and adults who were underweight. The study also found that the overall incidence of accidental arterial puncture in the entire study population was significantly higher when ultrasound guidance was not used. However, it was not a randomized controlled trial [11]. On the other hand, another study reported a high incidence of arterial puncture despite USG during internal jugular vein cannulation by surgical trainees [12].

If a carotid artery catheter gets placed inadvertently, it should not be removed immediately; rather, its position should be confirmed by CT angiography. The American Society of Anesthesiologists guidelines state that when inadvertent cannulation of an arterial vessel occurs, the catheter should be left in place, and immediate consultation with a vascular surgeon, an interventional radiologist, or a general surgeon should be sought to plan catheter removal [13]. Treatment should be guided based on existing resources and expertise, and should be individualized based on a number of factors, e.g., patient comorbidities, anticoagulation status, central venous catheter diameter, site of injury, time taken for injury to be recognized, etc.

Treatment options include open surgical exploration and vascular repair or percutaneous closure devices. Endovascular repair is particularly useful for patients with significant comorbidities in whom general anesthesia is not preferred, and for subclavian artery injuries that are difficult to access surgically [14,15]. Open surgical management is recommended when the injury is recognized more than 4 h after cannulation [15]. Our patient presented after 72 hours, so open surgical plan was opted.

The catheter was pulled out in the operating room, with a ready backup for open surgical intervention and repair if required. However, it was safely removed without any complications, i.e., bleeding, embolism, or vessel thrombosis, which was confirmed by a post-removal Doppler.

## Conclusions

This case demonstrates that if the catheter tip on X-ray of the right IJV vascular access appears to cross the mediastinum to the left, resembling a mirror image of left IJV placement, it may actually lie in the arch of the aorta, which can be confirmed by CT angiography. It is likely that this complication occurred because the placement was not image-guided, the backflow check was not interpreted carefully by the operator, and a post-procedure X-ray was not performed to verify its position. Taking these precautions into account before, during, and after placement may avoid these complications. Because of delayed presentation, open surgical exploration was opted for; however, the catheter could be removed without any complications or the need for surgery.

## References

[1] Bannon MP, Heller SF, Rivera M (2011). Anatomic considerations for central venous cannulation. Risk Manag Health Policy.

[2] Sato O, Tada Y, Sudo K, Ueno A, Nobori M, Idezuki Y (1986). Arteriovenous fistula following central venous catheterization. Arch Surg.

[3] Carr M, Jagannath A (1986). Hemopericardium resulting from attempted internal jugular vein catheterization: a case report and review of complications of central venous catheterization. Cardiovasc Intervent Radiol.

[4] Fangio P, Mourgeon E, Romelaer A, Goarin JP, Coriat P, Rouby JJ (2002). Aortic injury and cardiac tamponade as a complication of subclavian venous catheterization. Anesthesiology.

[5] Filograna L, Calcagni A, Rossi G, Di Stefano C, Beninati E, Collura A, Floris R (2019). Internal jugular vein agenesis: a rare vascular abnormality and incidental finding. A case of internal jugular vein agenesis in a 52-years old male. Radiol Case Rep.

[6] Kulvatunyou N (2003). Internal jugular vein and anatomic relationship at the root of the neck. Anesth Analg.

[7] Oliver WC Jr, Nuttall GA, Beynen FM, Raimundo HS, Abenstein JP, Arnold JJ (1997). The incidence of artery puncture with central venous cannulation using a modified technique for detection and prevention of arterial cannulation. J Cardiothorac Vasc Anesth.

[8] Reuber M, Dunkley LA, Turton EP, Bell MD, Bamford JM (2002). Stroke after internal jugular venous cannulation. Acta Neurol Scand.

[9] McGee DC, Gould MK (2003). Preventing complications of central venous catheterization. N Engl J Med.

[10] Maietta PM (2012). Accidental carotid artery catheterization during attempted central venous catheter placement: a case report. AANA J.

[11] Maddali MM, Arun V, Wala AA, Al-Bahrani MJ, Jayatilaka CM, Nishant AR (2016). Accidental arterial puncture during right internal jugular vein cannulation in cardiac surgical patients. Ann Card Anaesth.

[12] Hameeteman M, Bode AS, Peppelenbosch AG, van der Sande FM, Tordoir JH (2010). Ultrasound-guided central venous catheter placement by surgical trainees: a safe procedure?. J Vasc Access.

[13] Apfelbaum JL, Rupp SM, Tung A, Connis RT (2020). Practice guidelines for central venous access: reply. Anesthesiology.

[14] Guilbert MC, Elkouri S, Bracco D (2008). Arterial trauma during central venous catheter insertion: case series, review and proposed algorithm. J Vasc Surg.

[15] Dixon OG, Smith GE, Carradice D, Chetter IC (2017). A systematic review of management of inadvertent arterial injury during central venous catheterisation. J Vasc Access.

